# Cutaneous relapse of primary breast diffuse large B-cell lymphoma

**DOI:** 10.1016/j.jdcr.2024.09.023

**Published:** 2024-10-15

**Authors:** Henry Jeon, Daniela Rizzo, Sapna Amin, Tyler Moss

**Affiliations:** a Midwestern University Arizona College of Osteopathic Medicine, Glendale, Arizona; b Clin-Path Associates, Phoenix, Arizona; c All Dermatology, Glendale, Arizona

**Keywords:** chemotherapy, dermatopathology, diffuse large B-cell lymphoma, immunohistochemistry, lymphoma, medical dermatology, non-Hodgkin’s lymphoma, oncology, primary breast lymphoma, relapse, skin cancer, stem cell transplant

## Introduction

Diffuse large B-cell lymphoma (DLBCL) is an aggressive neoplasm of large B lymphoid cells characterized by a histologically diffuse growth pattern.^1^ It is the most prevalent form of non-Hodgkin lymphoma globally, often seen in older individuals, and is typically identified by the presence of rapidly proliferating tumors in 1 or more lymph nodes and extranodal sites upon initial presentation.^1^ Although the landscape of DLBCL treatment is quickly evolving, the current standard of care remains rituximab plus cyclophosphamide, vincristine, doxorubicin, and prednisone.^2^

Approximately one-third of DLBCL cases originate in extranodal organs, with common sites including the gastrointestinal tract, thyroid, testis, breast, and skin.^3^ Primary breast lymphoma constitutes less than 3% of extranodal lymphomas, DLBCL being the predominant histological subtype.^3^ The incidence of primary breast lymphoma has steadily increased over the past 4 decades. Despite this trend, there has been an improvement in the 5-year overall survival and progression-free survival rates, currently standing at approximately 79% and 67%, respectively.^4^

DLBCL demonstrates a recurrence rate of approximately 40%, with the majority of recurrences arising within the initial 3 years after immunochemotherapy.^5^ Primary breast DLBCL (PB-DLBCL), in particular, is predominantly associated with recurrence in the breast and central nervous system.^4^ Only one-third of DLBCL relapses occur without involvement of the primary site, and exclusive cutaneous relapse is rare.^6^

We report a case of cutaneous DLBCL of the upper extremity 4 months postremission of PB-DLBCL.

## Case report

We report a 61-year-old female patient who presented for a bumpy, nontender, mildly pruritic rash on her right lateral shoulder. She first noticed the lesion 1 month ago and reported a gradual increase in size. The patient denied trauma, insect bites, or a history of similar lesions. She denied fever, night sweats, weight loss, or other constitutional symptoms.

Past medical history was significant for a biopsy proven left-sided PB-DLBCL, germinal center-like with positive staining for BCL2, BCL6, myc, and Ki-67 index 90% 10 months ago. Positron emission tomography (PET) scan at the time of diagnosis of PB-DLBCL was negative for metastatic disease and bone marrow biopsy was negative for abnormalities. The PB-DLBCL was successfully treated with rituximab plus cyclophosphamide, vincristine, doxorubicin, and prednisone followed by rituximab, mesna, and etoposide, and a repeat PET scan 6 months after the initial diagnosis revealed no evidence of disease. Additional medical history included rheumatoid arthritis and hypertension. Social history was significant for a 40-pack-year smoking history, quit 15 years ago.

**Fig 1:**
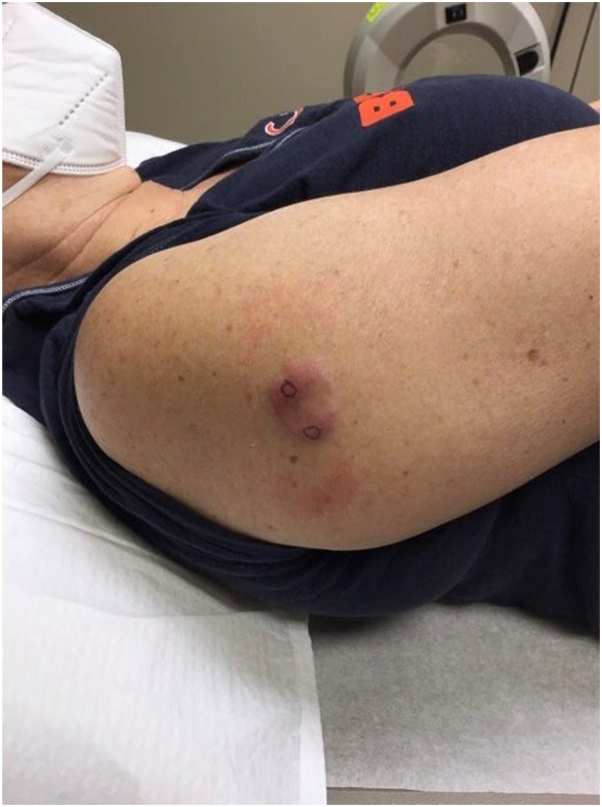
Cutaneous diffuse large B-cell lymphoma of right lateral shoulder.

Local examination revealed a 2.5 × 1.9 cm pink to mildly erythematous, nonscaly, indurated plaque with asymmetric borders on the right lateral shoulder (Fig 1). Two 4 mm punch biopsies were performed and were sent for histopathology with referral to oncology for PET scan. Differential diagnoses included granuloma annulare, cutaneous lymphoma, and annular elastolytic granuloma. Laboratory workup was unremarkable.

**Fig 2:**
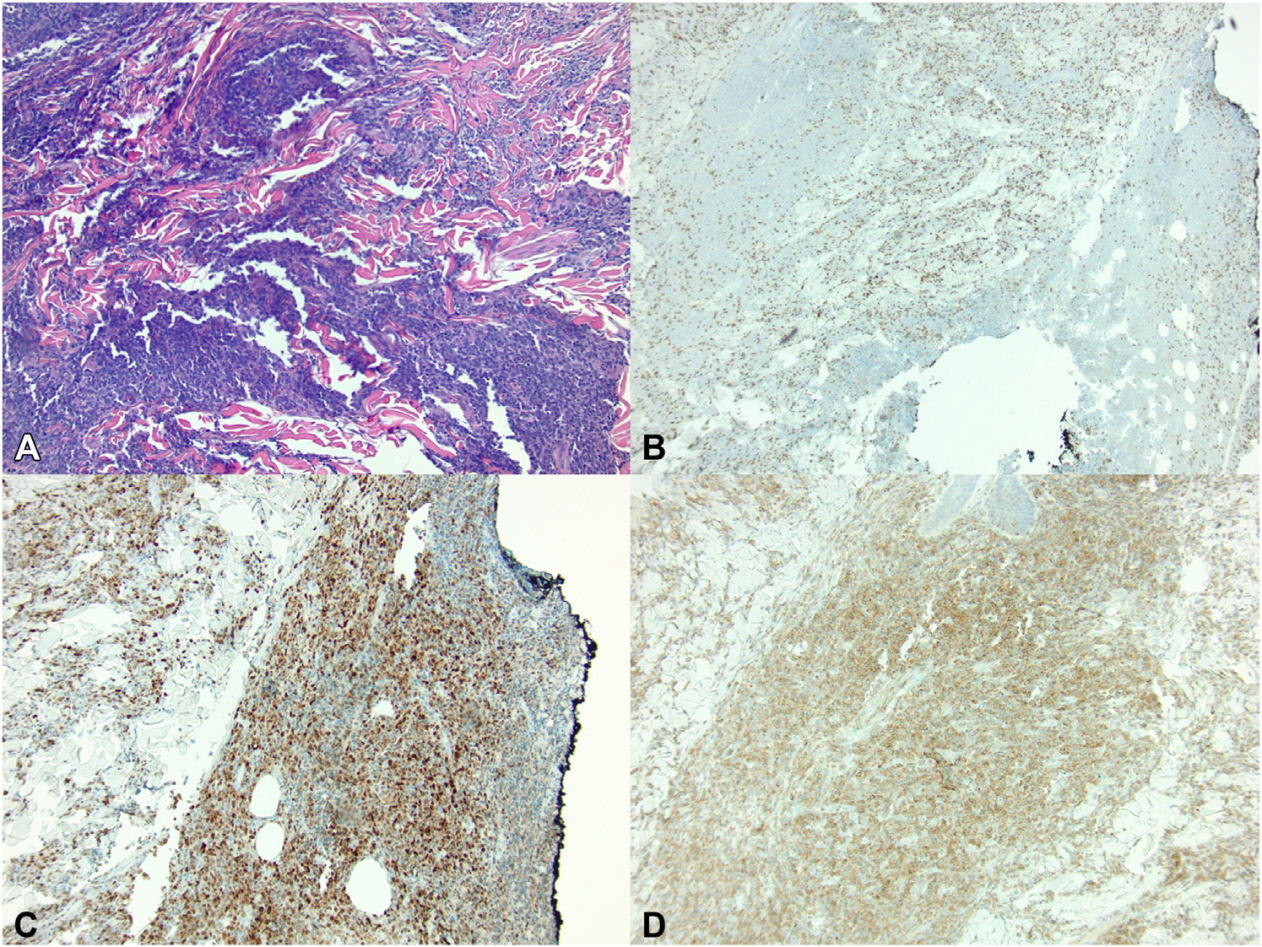
Histopathology of the cutaneous diffuse large B-cell lymphoma. **A,** Dense, diffuse lymphoid infiltrate involving the dermis (hematoxylin-eosin stain; original magnification ×10). **B,** CD3 highlights background B cells (original magnification ×4). **C,** Positive expression of MUM-1 (original magnification ×10). **D,** Neoplastic cells expressing BCL-2 (original magnification ×10).

Dermatopathology findings revealed lymphoid infiltrate involving the entire dermis positive for CD20 and paired box gene 5, with CD3 highlighting background B cells (Fig 2). The B cells co-expressed BCL2 (>50%) and MUM-1 (>30%) and were negative for CD10 and BCL-6 (variable, <30%). Ki-67 proliferative index was approximately 90%. The diagnosis of cutaneous DLBCL was made, consistent with the patient’s past primary lesion of the breast, 4 months postremission.

PET scan revealed increased uptake in a subcutaneous nodule in the right upper arm, a small subcutaneous nodule in the right face, and a small right lower lobe nodule. Magnetic resonance imaging of the brain revealed no evidence of disease.

Rituximab with ifosfamide, carboplatin, and etoposide (RICE) was initiated for the recurrent DLBCL with 2 courses. This was followed by carmustine (Bischloroethyl nitrosurea), etoposide, cytarabine, and melphalan (BEAM) with autologous stem cell transplant (ASCT) 2 months later, and a repeat PET scan 5 months post-ASCT revealed no evidence of disease.

## Discussion

There are limited data available on the prevalence and clinical features of secondary cutaneous DLBCL.^7^ To our knowledge, only 5 cases of recurrent cutaneous metastasis of a primary breast DLBCL have been described in the literature.^4^ However, it remains unclear whether these reported cases occurred in the presence of the original insulting lesion.

Cutaneous DLBCL typically presents as a localized red or violaceous papule, nodule, or plaque. Whereas primary cutaneous lesions often present in a solitary or localized fashion, secondary disease may present in a diffuse manner.^8^ Prognosis varies depending on the location of the lesion, with cutaneous DLBCL of the lower extremities having a poor prognosis at 5-year survival of 50%. Staining for multiple myeloma oncogene 1 may be performed to distinguish leg-type DLBCL compared to other forms, as positive multiple myeloma oncogene 1 staining indicates poor prognosis.^8^ Prognosis of secondary cutaneous DLBCL is not well described in literature but secondary DLBCL overall has poor long-term outcomes.^9^

The differential list for cutaneous DLBCL includes granulomatous diseases including granuloma annulare and annular elastolytic granuloma, and other malignancies including cutaneous pseudolymphoma and follicular lymphoma. Biopsy and histopathology are essential in diagnosis, and recommended staining panels for DLBCL include CD3, CD20, Ki-67, CD10, BCL6, multiple myeloma oncogene 1, BCL2, and myc.^8^ In addition, paired box gene 5 staining may be used to assist in establishing B-cell lineage, with a study revealing 100% detection in patients with DLBCL.^8^

Primary cutaneous DLBCL may be managed with radiation therapy, excision, chemotherapeutics, or intralesional corticosteroids. In our patient, the relapsing characteristic of the cancer and diffuse location of lesions indicated a more extensive treatment. BEAM-ASCT may be utilized in relapsed non-Hodgkin’s lymphomas, with complete remission rates nearing 78%. Studies revealed that achieving a complete response with a course of RICE prior to ASCT leads to improved progression-free survival rates compared to alternative pre-ASCT treatments in patients with relapsed or refractory DLBCL.^10^

## Conclusion

This is a rare case of secondary cutaneous DLBCL occurring post-remission of PB-DLBCL, successfully managed with RICE and BEAM-ACST. Prompt histopathologic evaluation of the lesion and advanced multimodal treatment strategies were necessary to manage an uncommon site of relapsing DLBCL, typically characterized by its ambiguous clinical presentation and poor prognosis.

## Conflicts of interest

None disclosed.

## References

[1] Li S., Young K.H., Medeiros L.J. (2018). Diffuse large B-cell lymphoma. Pathology.

[2] Poletto S., Novo M., Paruzzo L., Frascione P.M.M., Vitolo U. (2022). Treatment strategies for patients with diffuse large B-cell lymphoma. Cancer Treat Rev.

[3] Chen S.Y., Ji M.M., Xu P.P., Zhao W.L. (2022). Primary extranodal diffuse large B-cell lymphoma: molecular features, treatment, and prognosis. Aging and Cancer.

[4] Deng J., Mi L., Wang X., Zhu J., Zhang C., Song Y. (2022). Clinical prognostic risk analysis and progression factor exploration of primary breast lymphoma. Hematology.

[5] Sarkozy C., Sehn L.H. (2018). Management of relapsed/refractory DLBCL. Best Pract Res Clin Haematol.

[6] Adams H.J., de Klerk J.M., Fijnheer R. (2016). Where does diffuse large B-cell lymphoma relapse?. J Comput Assist Tomogr.

[7] Lee W.J., Won K.H., Won C.H. (2016). Secondary cutaneous diffuse large B-cell lymphoma has a higher international prognostic index score and worse prognosis than diffuse large B-cell lymphoma, leg type. Acta Derm Venereol.

[8] Lima M. (2015). Cutaneous primary B-cell lymphomas: from diagnosis to treatment. An Bras Dermato.

[9] El-Galaly T.C., Cheah C.Y., Bendtsen M.D. (2018). Treatment strategies, outcomes and prognostic factors in 291 patients with secondary CNS involvement by diffuse large B-cell lymphoma. Eur J Cancer.

[10] Kewalramani T., Zelenetz A.D., Nimer S.D. (2004). Rituximab and ICE as second-line therapy before autologous stem cell transplantation for relapsed or primary refractory diffuse large B-cell lymphoma. Blood.

